# Enhancement of Skin Delivery of Drugs Using Proposome Depends on Drug Lipophilicity

**DOI:** 10.3390/pharmaceutics13091457

**Published:** 2021-09-13

**Authors:** Himanshu Kathuria, Harish K. Handral, Saera Cha, Diep T. P. Nguyen, Junyu Cai, Tong Cao, Chunyong Wu, Lifeng Kang

**Affiliations:** 1 Department of Pharmacy, National University of Singapore, Singapore 117543, Singapore; himanshukathuria01@u.nus.edu (H.K.); a0113696@u.nus.edu (S.C.); a0126507@u.nus.edu (D.T.P.N.); 2 Stem Cell Bioprocessing, Bioprocessing Technology Institute, A*STAR, Singapore 138668, Singapore; harishhandral89@u.nus.edu; 3 School of Pharmacy, Faculty of Medicine and Health, University of Sydney, Sydney, NSW 2006, Australia; junyu.cai@sydney.edu.au; 4 Faculty of Dentistry, National University of Singapore, Singapore 119085, Singapore; dencaot@nus.edu.sg; 5 Key Laboratory of Drug Quality Control and Pharmacovigilance, China Pharmaceutical University, Nanjing 210009, China

**Keywords:** proposome, ibuprofen, tofacitinib, lidocaine, rhodamine, transdermal delivery

## Abstract

The study aims to investigate the propylene glycol-based liposomes named ‘proposomes’ in enhancing skin permeation of drugs with different physicochemical properties. Ibuprofen, tofacitinib citrate, rhodamine B, and lidocaine were loaded into proposomes. These drug formulations were analyzed for particle size, zeta potential, polydispersity index, entrapment efficiency, and in vitro skin permeation. The confocal laser scanning microscopy was performed on skin treated with calcein and rhodamine B laden proposomes. The transdermal delivery relative to physicochemical properties of drugs such as logP, melting point, molecular weight, solubility, etc., were analyzed. We tested the safety of the proposomes using reconstructed human skin tissue equivalents, which were fabricated in-house. We also used human cadaver skin samples as a control. The proposomes had an average diameter of 128 to 148 nm. The drug’s entrapment efficiencies were in the range of 42.9–52.7%, translating into the significant enhancement of drug permeation through the skin. The enhancement ratio was 1.4 to 4.0, and linearly correlated to logP, molecular weight, and melting point. Confocal imaging also showed higher skin permeation of calcein and rhodamine B in proposome than in solution. The proposome was found safe for skin application. The enhancement of skin delivery of drugs through proposomes was dependent on the lipophilicity of the drug. The entrapment efficiency was positively correlated with logP of the drug, which led to high drug absorption.

## 1. Introduction

Transdermal delivery has been widely studied due to its many advantages, such as avoiding first-pass metabolism, reducing side effects, improving patient compliance, and increasing bioavailability. The three ways in which a drug molecule could penetrate skin are intracellular, intercellular, and follicular pathways. However, regardless of which pathway the drug takes, the major challenge in this transdermal mode of drug delivery is the presence of the outermost stratum corneum (SC), made up of corneocytes inter-dispersed in the lipid matrix. SC acts as a primary barrier, only allowing small and lipophilic molecules to pass through [1].

Various methods have been developed over the past few decades to enhance drug permeation through the skin [2,3]. In recent years, new types of nanovesicles have been reported for transdermal drug delivery, e.g., glycerosomes [4], glycethosomes [5], hyalurosomes [6], and proposomes [7]. These nanovesicles are classified as penetration enhancer-containing vesicles (PEVs) [8]. Apart from transdermal drug delivery, PEVs have also been used to deliver therapeutic compounds into human through gastrointestinal tract [9], oral cavity [10], and nail [11]. In addition, various other nano systems including liposomes have also been developed for skin delivery, such as hybrid liposomes [12], hybrid nanoparticle [13], and surface functionalized liposomes [14]. Surface functionalization of liposomes and nanoparticles can be helpful to target specific tissues, cells or appendages in skin.

Among the PEVs, liposomes containing propylene glycol (PG) have been shown to improve drug delivery via intercellular pathways [15]. It has been shown that PG interacts with SC intercellular lipids [16] and can be used as a suitable solvent to deliver drug through skin [17,18]. PG does not cause skin irritation and, being less volatile than ethanol, has better stability due to less evaporation during storage [19]. A report showed the ethanol treatment could cause significant morphological changes in frog skin and separation of the skin layers. In contrast, the treatment with 20% PG only caused swelling of top skin layers while significantly enhancing skin permeation [20].

**Figure 1:**
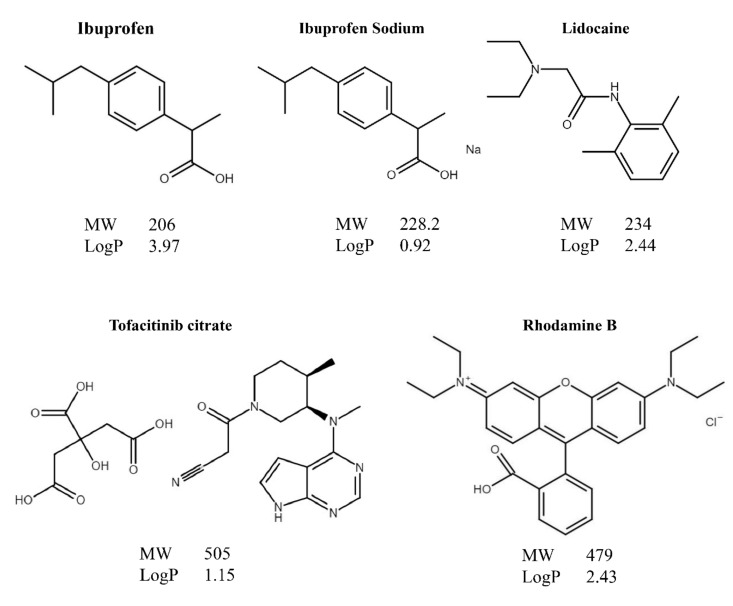
Chemical structure, molecular weight (MW) and logP of model drugs loaded into proposomes to study the skin delivery.

Recently, we characterized PG-based liposomes systematically and named it as proposome from a combination of ‘prop’ of propylene glycol and ‘osome’ of liposome [7]. It was found that proposome can enhance the skin deposition and permeation of tofacitinib citrate, a challenging molecule to be delivered through the skin. This has motivated us to determine whether proposome can enhance the skin permeation of other drugs and the general trend of its capability of enhancing drug permeation through skin. To this end, ibuprofen, tofacitinib citrate, lidocaine, and rhodamine B were investigated. These four molecules have different physicochemical properties, mainly, the molecular weight (MW) and logP (Figure 1).

Topical ibuprofen and its sodium salt are used in pain and inflammation in rheumatism and other musculoskeletal diseases [21]. Tofacitinib citrate has been used for many inflammatory skin diseases, such as psoriasis [22], while lidocaine has been a local anesthetic agent [23]. Rhodamine B, a fluorescence probe, is commonly used for histological study in transdermal experiments [24]. These drugs have been studied for topical use previously [21,22,23,24,25], but not particularly for proposome. Current commercial products of these drugs often contain multiple penetration enhancers [26,27], which are known to cause skin irritation [19] and requires a high amount of a drug inside the product to achieve therapeutic effects. Delivery of these drugs, utilizing proposome, acts as an alternative with reduced side effects, since PG is safer than other alcohol types [28].

In vitro skin permeation experiments are generally performed to detect the amount of the drug permeated through the skin over time. They can provide information on drug permeation profiles through characterizing the effectiveness of the delivery system [29]. In addition to skin permeation, safety assessments are a critical part of new formulation developments for topical and transdermal drug delivery. Cadaveric skin has a long history of use for in vitro efficacy and safety testing of topical drug products. However, cytotoxicity tests are impossible with cadaveric skin, such as assessing inflammatory markers, which require viable cells or tissue models. To this end, in vitro skin models were developed by researchers using advanced bioengineering techniques. In this study, we used a bioengineered skin model to assess the formulation’s safety, which was produced in-house with the vascularized dermis. The bioengineered skin mimics the native skin tissue with promising performance to test topical formulations.

In this study, we prepared proposomes containing low molecular drugs of different physicochemical properties using a cold mixing process. The particle size, polydispersity index (PDI), and zeta potential of the proposome were determined using dynamic light scattering and zeta potential measurement. An in vitro skin permeation test was performed to study the effectiveness of the proposomes in enhancing the skin penetration of the drugs. Based on the experimental results, we established the correlation between the physicochemical properties of the drugs and their effects on skin permeation and skin retention of the drugs. Afterward, the skin penetration of the proposome was investigated using confocal microscopic imaging. Lastly, reconstituted human skin (RHS) samples were prepared to study the toxicity of the proposome. The tissue viability was assessed using colorimetric measurement, while the skin irritation potential was tested by measuring the levels of cellular interleukin 1α and interleukin 8.

## 2. Materials and Methods

### 2.1. Materials

Soya phosphatidylcholine (SPC) was obtained from the Lipoid (Steinhausen, Switzerland), under the brand name of Phospholipon 90. The suppliers of other reagents were: PG (Chempure, Singapore), dialysis tubing (Thermo Scientific, Waltham, MA, USA), ibuprofen and ibuprofen sodium (Sigma, Singapore), tofacitinib citrate (Sigma, Singapore), rhodamine B (Sigma, Singapore), calcein (Sigma, Singapore), lidocaine (Tokyo Kasei, Tokyo, Japan), phosphate buffered saline (PBS) (Vivantis, Singapore). Polyethylene glycol-4-arm, succinimidyl glutarate, bovine fibrinogen, bovine thrombin and calcium chloride were purchased from Sigma-Aldrich, Singapore. Growth medium for culturing keratinocytes, fibroblasts, pericytes and human dermal microvascular cells were purchased from PromoCell, Heidelberg, Germany. ThinCert culture ware and tissue culture flasks were purchased from Greiner Bio-One, Kremsmünster, Austria. Water was purified by using Milli-Q system (Millipore, Billerica, MA, USA).

### 2.2. Preparation of Proposome

**Figure 2:**
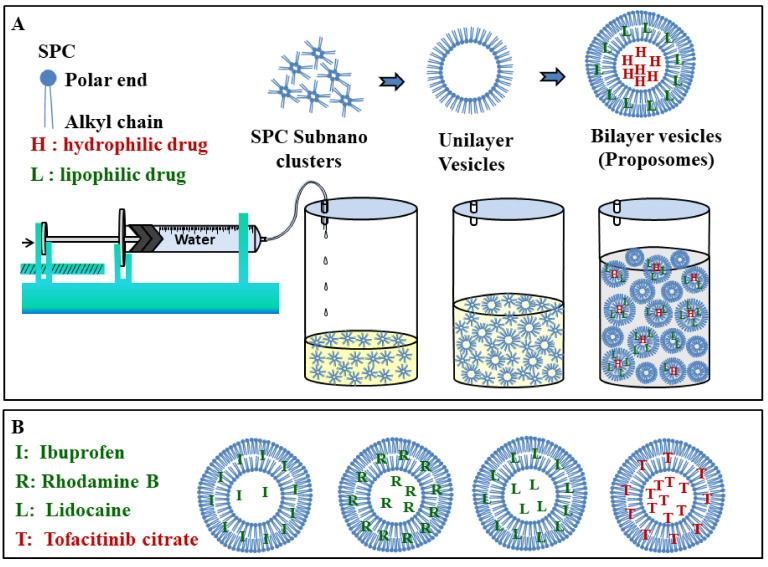
(**A**) Schematic of preparation process. The dropwise addition of water into PG (also SPC and drug) with constant magnetic stirring causes proposome formation by rearrangement of SPC molecules to the vesicular structure. (**B**) Probable distribution of drug molecules in the proposomes. Lipophilic drugs are more likely in bilayer and less likely in the core while it is reverse for hydrophilic drugs.

Proposome was prepared using a cold mixing process as shown in the schematic in Figure 2 [7]. Firstly, SPC was dissolved with drug in PG under constant stirring, followed by the continuous dropwise addition of Milli-Q water through a syringe pump (LSP10-1B-10 Channels Syringe Pump, Longer Precision Pump Co., Ltd., Baoding, China) at a constant rate of 1 mL/min and magnetic stirring, at 1500 revolution per minute (rpm), under room temperature. Water was added to the vortex axis to ensure even mixing, and the system was kept closed throughout to prevent evaporation. The size of the container and the magnetic bead was kept constant to minimize variation. The final product was stirred for an additional 30 min for stability. All proposomes were prepared with 1% *w*/*v* SPC, 30% *v*/*v* PG, and a drug (ibuprofen, tofacitinib citrate, rhodamine B, calcein, and lidocaine) concentration of 5.64 mM for each.

### 2.3. Proposome Characteristics

The dynamic light scattering technique was employed to study particle size, polydispersity index (PDI), and zeta potential of various formulations using Zetasizer Nano ZS90 (Malvern Instruments, Malvern, UK). Proposomes were diluted with 30% PG in the ratio of 1:20 and equilibrated at 25 °C before measurement. All measurements were conducted in triplicates at an automatic measurement mode.

### 2.4. Entrapment Efficiency and Drug Loading

Entrapment efficiency (%) was obtained using a dialysis bag made of a regenerated cellulose membrane with molecular cut-off 3500 Da (Thermo Scientific SnakeSkin Dialysis Tubing, Waltham, MA, USA). The dialysis membrane was cut into ~3 cm length and clipped on both sides after putting the formulation into the bag. Moreover, 0.5 mL of the proposome was dialyzed against Milli-Q (100 mL) water, with constant stirring, using a magnetic bead at 75 rpm for 1–1.5 h, depending on the time required for at least 90% of the drug release from the free drug solution. The filtrate was analyzed using high performance liquid chromatography (HPLC) to determine the amount of the free drug. Entrapment efficiency was then calculated using Equations (1) and (2).

(1) $$\mathrm{Entrapment}\;\mathrm{efficiency}\;\left(\%\right)=\frac{W_{total}-W_{free}}{W_{total}}\times 100$$

(2) $$\mathrm{Drug}\;\mathrm{loading}\;\left(\%\right)=\frac{W_{total}-W_{free}}{W_{total\;SPC}}\times 100$$

where $W_{total}$ = total amount of the drug incorporated into proposome, $W_{free}$ = amount of the free drug obtained from the filtrate after dialysis and $W_{total\;SPC}$ = total weight of SPC used in the proposome formulation.

### 2.5. In Vitro Skin Permeation

#### 2.5.1. Skin Permeation Method

Human cadaver skin samples used in the study were via a donation, from a 57-year-old Caucasian female, obtained from Science Care (Phoenix, AZ, USA), without an identifier and, hence, exempted from ethical review. Dermatomed thigh skin (epidermis and partial dermis) with thickness of 250–300 µm was used for the skin permeation study. Vertical Franz diffusion cell was used for the study, with an effective diffusion area of 0.25 cm^2^. Skin was placed between the donor and receptor compartment, with the SC side facing the donor compartment. A dose of 50 µL of proposome solution was added to the donor compartment and 4 mL of PBS (pH 7.4) was placed inside the receptor compartment to maintain sink condition. A brief discussion considering sink conditions can be found in Supplementary Materials S1, with the solubility data of each drug found in literature [30,31,32,33]. The system was placed under constant stirring of 250 rpm at 32 °C to mimic in vivo skin environment. Parafilm was used to cover both the donor compartment and sampling port to prevent evaporation throughout the experiment. Samples of 1 mL were collected from the receptor compartment at 1, 2, 4, 8, 12, and 24 h. After each sampling, 1 mL of fresh PBS was added to the receptor compartment to maintain constant volume. Samples were then centrifuged at 10,000 rpm for 5 min, and the supernatant was analyzed with an LC-20AD HPLC machine (Shimadzu, Kyoto, Japan). Control was also prepared by dissolving the same amount of drug in 30% PG solution without SPC.

After 24 h of skin permeation, the skin samples were removed and washed with PBS to remove the free drug on the skin surface. The skin sample was processed and analyzed using the method we reported previously [7,34]. Briefly, the washed skin samples were digested by proteinase K solution. Then the digested skin samples were processed and analyzed using HPLC.

#### 2.5.2. Skin Permeation Parameters

The skin permeation results were processed to generate the following parameters: cumulative permeation (CP), and area under the curve (AUC) from proposomes. The AUC was employed to represent the total drug permeation through skin across time. The formulation process and drug molar concentrations were the same for all proposomes. Further, enhancement and enhancement ratio were calculated for CP and AUC of proposomes compared to CP and AUC of the control (free drug in 30% PG). The CP enhancement (EN_CP_) (Equation (3)), CP enhancement ratio (ER_CP_) (Equation (4)), AUC (Equation (5)), AUC enhancement (EN_AUC_) (Equation (6)), and AUC enhancement ratio (ER_AUC_) (Equation (7)) were calculated using the following equations:

(3) $$CP\;Enhancement\;\left(EN_{CP}\right)=A_{proposome\;\;}-A_{Control\;\;}$$

(4) CP Enhancement ratio ERCP=Aproposome  AControl  

where, $A_{proposome\;}$ and $A_{Control\;}$ were the cumulative amounts permeated in 24 h for proposomes and control, respectively.

(5) $$Area\;Under\;Curve\;\left(AUC\right)=\sum \left(X_{2}-X_{1}\right)\left(Y_{1}+Y_{2}\right)/2$$

where, $X_{\;}$ was time and Y was the cumulative permeation at the time.

(6) $$AUC\;Enhancement\;\left(EN_{AUC}\right)=AUC_{proposome\;\;}-AUC_{Control\;\;}$$

(7) AUC Enhancement ratio ERAUC=AUCproposome  AUCControl  

where, $AUC_{proposome}$ and $AUC_{control}$ were AUC_0→24h_ for proposome and control respectively for 24 h.

The EN_CP_ presents the increase in absolute drug amount permeated through the skin from proposomes compared to the drug amount permeated from the free drug in 30% PG (control). The ER_CP_ is the ratio that shows the factor by which drug permeation is enhanced in proposomes compared to the control. The AUC presents the overall drug permeation profile over 24 h. The EN_AUC_ presents the increased AUC from proposomes compared to the control. The ER_AUC_ is the ratio that shows the factor by which the AUC is enhanced.

### 2.6. Confocal Laser Scanning Microscopy (CLSM)

CLSM was used to visualize the pathway and depth at which proposomes penetrated skin. Proposomes loaded with 5.64 mM rhodamine B (MW = 479.0, logP = 2.43) and calcein (MW = 622.5, logP = 1.61) were applied on skin mounted in the Franz diffusion cell. After 24 h, the skin samples were removed and surfaces were washed with deionized water thoroughly before they were examined under the microscope. The images were obtained using a Nikon A1 plus Ti Microscope (Nikon, Singapore).

### 2.7. Fabrication of Reconstructed Human Skin (RHS)

**Figure 3:**
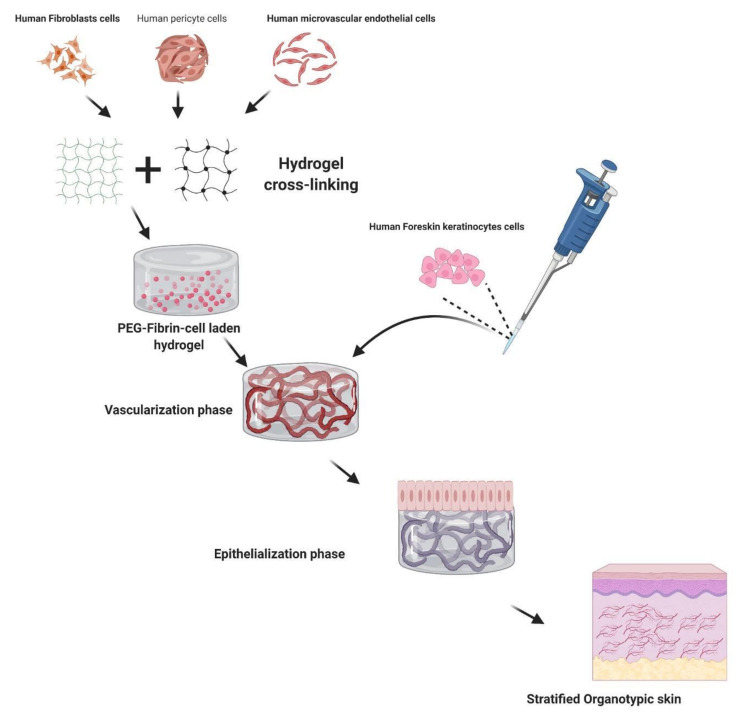
The schematic representation of fabricating RHS from human cells and hydrogels.

Human foreskin keratinocytes, human dermal fibroblasts, human pericytes, and human dermal microvascular endothelial cells were purchased from PromoCell. All cells were cultured and maintained as recommended by the manufacturer. The RHS preparation process is illustrated in Figure 3. Fibrinogen from bovine plasma, polyethylene glycol-4-arm, and succinimidyl glutarate terminated were used to fabricate fibrin gels, following previously published reports [35,36]. Briefly, fabrication of PEG-fibrin gels was conducted by mixing PEG and fibrinogen in a ratio of 40:1, making the final concentration of fibrinogen and PEG to 10 mg/mL and 0.25 mg/mL, respectively. This mixture was incubated at 37 °C for 20 min to 30 min. Later, optimized cell density was suspended in an appropriate volume of media in PEG-fibrinogen mixture. Parallelly, thrombin was used for the gelation of PEG-fibrinogen using 40 mM calcium chloride (CaCl_2_) in a ratio of 3:1. A PEG-fibrin scaffold consisting of the cells was fabricated and cultured for 10 days to form pre-vascularized beds. Later, topical seeding of keratinocytes was performed and allowed to undergo standardized stages of skin organotypic culture, i.e., 2 weeks of an air–liquid interface to attain the stratification. During the culture period, 3D cultures were fed with optimized media [37] with renewal every 24 h, which supported the formation of skin tissues. After the air-liquid interface phase, the RHS tissues were harvested for further processing.

### 2.8. Safety Assessment of Proposomes

#### 2.8.1. Skin Histology

The human cadaver skin samples and RHS samples were treated with 30% PG, blank proposomes and TC-proposomes for 24 h, respectively. Para-formaldehyde (4%) fixed tissue samples were tissue processed using an automatic tissue processor (Shandon Citadel 2000, Thermo Fisher, Waltham, MA, USA) in which tissue samples were treated with increasing concentrations of alcohol, three different incubations in xylene and finally in molten paraffin for different durations of time, following a standard protocol. Moving towards the embedding section, tissues were embedded in paraffin wax poured in metal block, which can hold tissue samples in appropriate position, and were placed on a cold plate to fix the tissue position. After 30–40 min of cooling, paraffin blocks were moved to 4 °C. A microtome machine was used to obtain thin sections (5 µm) of paraffin embedded tissue samples. Thin sections were collected onto glass slides, which were taken over for hematoxylin and eosin (H&E) staining, by following a standardized protocol. All stained sections were imaged using a microscope (Nikon SMZ25, Tokyo, Japan) and processed with the Nikon imaging software (NIS-Element analysis D 4.30.00).

#### 2.8.2. Tissue Viability Using MTS Assay

The RHS samples were treated with PBS, 30% PG, blank proposomes, TC-proposomes and sodium dodecyl sulfate (SDS) for 24 h, respectively. After treatment, the skin samples were washed gently with PBS to remove unbound chemicals. The washed skin tissues were assessed for their viability using MTS assay (CellTiter 96^®^ Aqueous One Solution Cell Proliferation Assay, Promega, Madison, WI, USA). 40 μL of MTS reagent was added on the top surface of the treated tissue and incubated for 4 h at 37 °C. The color developed in MTS suspension was eluted in a separate 96-well plate and absorbance was recorded at 490 nm using a plate reader (M200 infinite, Tecan, Männedorf, Switzerland). The skin tissues treated with distilled water were considered as the control. The absorbance values of the test samples were compared with controls.

#### 2.8.3. Cytokine Assay to Study Inflammatory Response

A quantitative sandwich immunoassay technique (Quantikine, R&D System, Abingdon, UK) was used to determine the extracellular expression of IL-1α and IL-8 in the supernatant collected after treatment with proposomes and controls for 24 h. The detection limit of IL-1α and IL-8 was 0.5 and 10 pg/mL, respectively. In brief, 500 μL of post-treated test samples were collected and frozen for further enzyme-linked immunosorbent assay (ELISA) experiments. Prior to the loading of collected samples, ELISA plates were coated with reagent diluent by following the instructions with the kit. After washing the pre-coated wells with a buffer, wells were loaded with collected samples and/or standards and incubated for 2 h at room temperature. The incubated plates were again washed, loaded with 200 μL of IL-1α or IL-8 conjugates, and further incubated for 1 h. The incubated mixtures were discarded, and plates were washed thrice 100 μL of horseradish peroxidase (HRP)-conjugated streptavidin were added to each well and incubated in dark for 20 min. A 50 μL volume of stock solution was added to each well and thorough mixing was ensured by gentle tapping. The optical density of each well was determined by immediately recording the absorbance at 450 nm using the plate reader. The concentrations of IL-1α or IL-8 in the samples were calculated using the calibration curve. The standard curves were recorded for each assay for both molecules. The values from specific control cultures were subtracted from the tests. The potential interference with each ELISA by the test chemicals was evaluated by assaying known amounts of each cytokine in the presence of appropriate concentrations of the test chemicals.

### 2.9. HPLC Method

Validated HPLC methods, adapted from other studies [38,39,40,41], were used to quantify the amount of drug entrapped in the proposome, permeated through the skin, as well as what was retained in the skin. The HPLC conditions for the analysis of different drugs have been shown in Supplementary Materials S2.

### 2.10. Statistical Analysis

Data collected were analyzed using Microsoft Office Excel 2010 (Microsoft, Redmond, WA, USA) and all the results are presented as mean ± standard deviation. SPSS (SPSS Inc, Chicago, IL, USA) was used to run Student’s *t*-test with alpha of 0.05 to test statistical significance. A *p*-value of <0.05 was considered statistically significant. The physicochemical properties, such as logP, solubility, melting point (mp), MW, etc., obtained from the online sources were linearly regressed to identify the relationship with skin permeation results. The regression coefficient (R^2^) above 0.8 was considered a good linear correlation, while R^2^ below 0.5 was considered a low or no linear correlation. The total drug amount delivered (sum of the drug permeated into the receptor compartment and amount retained in the skin), and drug amount permeated into the receptor compartment were separately studied.

## 3. Results and Discussion

### 3.1. Proposome Characteristics

Table 1 shows the various parameters studied for proposomes. The average size of most of the proposomes ranged from 140 to 150 nm, except for rhodamine B, which has a smaller size of 128 nm. Regardless of the drug loading, all proposomes had an average size of less than 300 nm, which was shown to increase drug delivery to deeper layers of the skin [42]. The smaller particle size of rhodamine B proposomes could be attributed to the ion–ion interaction between the positively charged quaternary amine in rhodamine B and the negatively charged phosphate group in SPC [43]. Such interaction brings the liposomal structure tighter, leading to a smaller size.

PDI has been an indicator for formulation homogeneity, and all values were less than 0.2, indicating narrow size distribution and size uniformity [44]. Zeta potential was another important parameter of proposomes, and it was between −32.5 and 11.8 mV. Compared to a blank proposome with a zeta potential of -28.7 mV, drug addition resulted in a change of the zeta potential. The difference in the zeta potential of various proposomes was due to physicochemical properties, especially pKa and ionization. It was observed that the more basic the drug, the more positive the zeta potential.

The encapsulation efficiency for various drugs in proposomes varied between 42% to 55%, and the mean value was 51.5%. It may seem low; however, increasing drug encapsulation is not the objective of current study. We kept the composition and drug molar ratios constant to compare the differences in the enhancement of drugs with different physicochemical properties through proposomes. On the other hand, the drug encapsulation can be enhanced potentially by varying the composition of the proposomes. For instance, the amount of SPC in proposome preparation may be increased to obtain high drug entrapment.

The lipophilic drugs, such as ibuprofen (logP = 3.97) showed a higher entrapment efficiency than hydrophilic drugs, such as tofacitinib citrate (logP = 1.15). For entrapment, lipophilic drugs with greater logP, such as ibuprofen (logP = 3.97), showed a considerably higher entrapment efficiency than those drugs with smaller logP, such as tofacitinib citrate (logP = 1.15). This could be because highly lipophilic drugs are entrapped within the lipid bilayer and, hence, show minimal leakage. In contrast, hydrophilic drugs tend to leak out to the outside aqueous medium to a greater extent [45]. Calcein loaded proposomes had an average diameter of 148 ± 1.80 nm, PDI 0.116 ± 0.019, and zeta potential −5.42 ± 3.60. The entrapment efficiency for calcein loaded proposomes was not performed due to difficulties in quantitative measurements.

The pH was also measured, shown together with drug pKa values [32,33,46,47] (Supplementary Materials S3). However, as PG is an organic solvent, it is difficult to interpretate the pH and degree of ionization in the 30% PG solution.

### 3.2. In Vitro Skin Permeation

**Figure 4:**
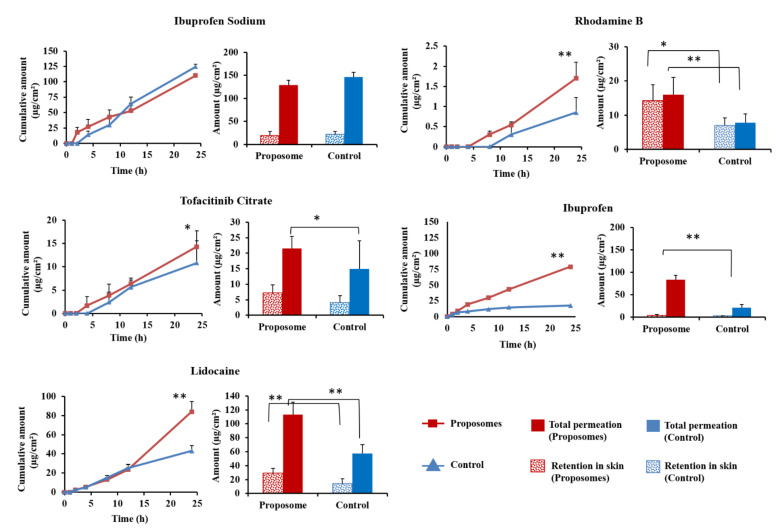
Skin permeation of proposomes and control for 24 h; drug retention in skin and the sum of permeated and retained. ** indicates statistically significant difference between proposome and control with *p*-value < 0.01. * indicates statistically significant difference with *p*-value < 0.05.

As shown in Figure 4, 24 h skin permeation for all proposomes, except for ibuprofen sodium, showed a higher amount of drug than control (*p* < 0.05). In the case of ibuprofen, proposome was able to increase its skin permeability at least four times compared to control. The total drug amount in the receptor compartment and drug retained showed a significant increase via proposomes compared to control. For ibuprofen sodium, the amount of drug permeated by proposome and control showed no statistical difference (*p* = 0.135). Incorporating ibuprofen sodium into the proposomes did not improve its permeability into the skin.

Proposome increased both drug permeation and drug retention in the skin for all drugs, excluding ibuprofen sodium. PG acts as a penetration enhancer that disrupts the SC intercellular lamellae and can cause the reversible redistribution of tight junctional proteins, thereby increasing drug skin permeability. When made into a liposome, it also acts as a vesicular carrier that bypasses the stratum corneum and releases the drug molecules into the deeper layer of skin [48].

Although ibuprofen sodium proposome showed similar results to control, the actual drug amount that permeated and retained was very high compared to ibuprofen proposomes (Figure 4). The ibuprofen sodium proposome had a comparable amount permeated when compared to ibuprofen proposome. This suggests that it already has a high permeation profile with 30% PG, and a further increase in permeation through proposome was negligible. This could mean that most of the drug permeation was due to free drug, and proposome did not play a significant role in the delivery. It has been shown that PG helps, primarily as a penetration enhancer for hydrophilic molecules rather than a carrier [49]. Thus, adding PG without making it into the liposomal structure was enough to improve drug permeation and skin retention of hydrophilic molecules, i.e., ibuprofen sodium.

We included the control for each drug in the in vitro skin permeation study, i.e., drug solution in 30% PG, where PG percentage and drug molar ratios were kept the same as those of proposomes. The contrast between 30% PG solution and 30% PG solution containing proposome can demonstrate the enhancement achieved by proposome. Blank liposomes with an externally added drug could have been a better control to study the effect of the proposomes, which, however, is not feasible, as the externally added drug could be passively loaded into the proposomes with time.

### 3.3. Correlation between the Physiochemical Properties and Skin Delivery of Drugs

The improvement in the skin permeation of drugs using proposomes is evident in Figure 4. However, we wanted to establish the correlation between skin permeation and physicochemical properties of loaded drugs. Rhodamine B and lidocaine have logP of 2.43 and 2.44, respectively, and the corresponding ratios (ER_CP_ and ER_AUC_) were also close to each other. This is interesting to note because rhodamine B (MW = 479.0) has twice the MW of lidocaine (MW = 234.3). Still, the difference in MW did not seem to influence the permeability, and only logP led to a significant change. Despite the large MW, rhodamine B containing proposome increased the skin delivery by 2.04 times, compared to the control. It was an indication that even drugs with relatively large MW can be delivered to the skin when loaded into proposome. Ibuprofen, being the most lipophilic drug of all, showed the highest ER_CP_ and ER_AUC._ The relatively low fold change in skin retention could be attributed to saturation of skin with the drug, leading to a greater drug amount being permeated through, rather than being retained.

**Figure 5:**
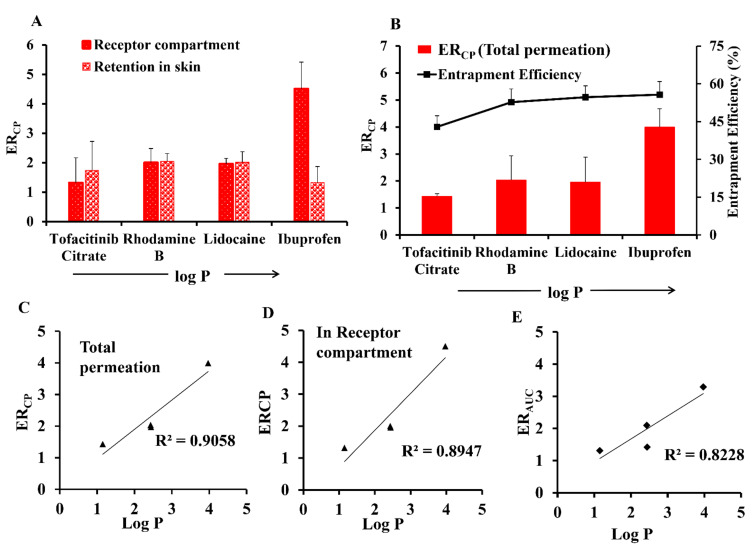
(**A**) Effect of logP of drugs delivered through proposomes on amount retained and amount permeated. (**B**) Effect of logP on entrapment efficiency and ER_CP_. (**C**) Linear regression of plot of logP vs ER_CP_ for total permeation. (**D**) Linear regression of plot of logP vs ER_CP_ for amount of drug permeated into receptor compartment. (**E**) Linear regression of plot of logP vs ER_AUC_. The linear regression analysis was based on ibuprofen, lidocaine, tofacitinib citrate, and rhodamine B, while ibuprofen sodium was excluded.

Figure 5 shows that drug entrapment efficiency was also correlated with logP and ER_CP_. The positive association between logP and ER_CP_ can be explained by entrapment efficiency. Previous studies on liposomes have also established that lipophilic drugs were better entrapped than hydrophilic drugs [45] and higher amount of entrapped drug means more drug can be carried to skin by proposome, therefore leading to greater drug permeation. It should also be noted that even a small increase in entrapment efficiency increased skin permeation significantly, showing that the proposome indeed had a major role in enhancing the drug delivery to the skin.

The physicochemical properties were the only independent variables affecting skin permeation parameters as other variables were kept constant in all formulations. However, both free drugs and encapsulated drugs in proposomes must be considered to correlate with skin permeation parameters. Based on skin permeation data of the drugs, the logP, mp, and MW showed linear correlation with skin permeation parameters (Table 2). The ibuprofen sodium was excluded, correlating with better comparison, and avoiding misinterpretation due to the sameness of ibuprofen and ibuprofen sodium.

The ER_CP_ presents an improvement in skin permeation by proposome, and it had a positive linear correlation with drug logP and entrapment efficiency (Figure 5). The ER_AUC_ also had a positive linear correlation with the logP (Table 2), while no linear correlation was observed with CP. In contrast, there was no linear correlation between ER_CP_ or ER_AUC_ with mp, while inverse linear correlation with CP, AUC, and EN_CP_ was observed. Similarly, the MW also showed an inverse linear correlation with CP, AUC, and EN_CP_, while no linear correlation with either ER_CP_ or ER_AUC_. Therefore, we could conclude that the mp and MW affected the skin permeation via proposomes in the same way. The ER_CP_ also showed a linear correlation to entrapment efficiency other than drug logP (Figure 5B), while no linear correlation was observed between entrapment efficiency and ER_AUC_. The correlation trend was similar for total drug permeation and drug permeation into the receptor compartment. The ER_CP_ based on both the amount permeated into the receptor compartment (Figure 5C) and total permeation (Figure 5D) were linearly related with logP. Similarly, ER_AUC_ was also linear with logP (Figure 5E).

As shown in Table 2, the different physicochemical properties have a distinct correlation with drug permeation parameters. This indicates that various physicochemical properties simultaneously affected drug skin permeation. Moreover, the absence of linear correlation between logP with either CP or EN**_CP_** also shows that more than one physicochemical property simultaneously affected skin permeation. The lidocaine and rhodamine B, despite having the similar logP showed different CP and EN**_CP_**. However, ER**_CP_** and ER_AUC_ for lidocaine and rhodamine B were not significantly different, which can be attributed to the normalization of other factors as both these parameters are calculated as ratios. For ratios (ER_CP_ and ER_AUC_), physicochemical factors (mp, solubility and MW) with the respective control would have been normalized as these factors do not affect encapsulation into proposomes. In contrast, the skin parameters calculated as ratios did not have linear correlation with mp and MW. This revealed that linearity of CP and EN**_CP_** with mp and MW represented the correlation due to free drug in formulation (unencapsulated drug outside proposomes), which follow passive absorption into the skin. In contrast, the ratios (ER_CP_ and ER_AUC_) linearity with logP truly represents the drug encapsulated in proposomes. It reveals that other absorption mechanisms also contribute to drug permeation via proposomes such as the follicular route. Therefore, we could conclude that for comparison of skin permeation parameters, the ratios (ER_CP_ and ER_AUC_) better represent an encapsulated drug in proposomes, while EN**_CP_** and CP better represent an unencapsulated drug.

### 3.4. Confocal Imaging of Treated Skin

**Figure 6:**
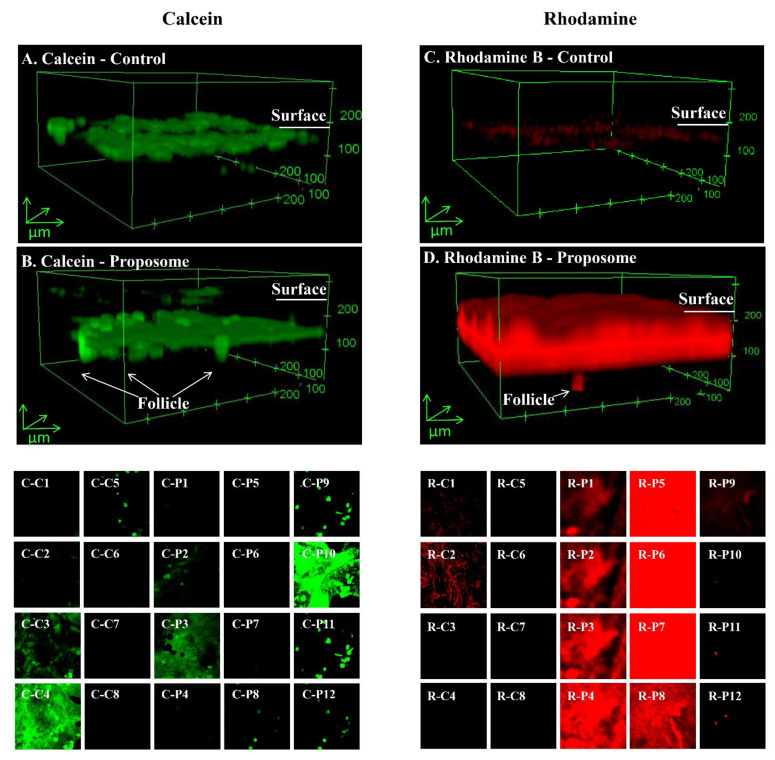
The confocal microscopic images showing fluorescent calcein control (**A**) /proposome (**B**) and rhodamine B (**C**) /proposome (**D**) penetration into human skin after 24 h treatment. Sliced images of calcein control (C-C1 to C8)/proposomes (C-P1 to P12, and of rhodamine B control (R-C1 to C8) and proposome (R-P1 to P12). Each slice is 20µm. The control was 0.2% *w*/*v* calcein or rhodamine B solution in 30% PG. The concentrations of calcein and rhodamine B were same for proposomes and control.

As shown in Figure 6, proposomes led to a greater rhodamine B and calcein deposited in the skin than the control. From the skin surface, proposomes also showed drug deposition in deeper regions of the skin, more than 100 µm from the surface, while in the control case, most of the drug remained on the skin surface. It can be observed that, other than the intercellular pathway, which is the usual pathway taken by the control (Figure 6A,C), proposomes also undertook the transfollicular route for drug delivery as indicated in Figure 6B,D. Indeed, several studies have also shown liposome capacity to aid drug delivery through the appendageal pathway [50]. It was found that proposomes help permeation of rhodamine B to a greater extent than calcein. This was again in line with the correlation between drug logP and ratios (ER_CP_ and ER_AUC_) due to higher drug entrapment within the proposome. Rhodamine B, being more lipophilic than calcein, would have higher entrapment efficiency.

The sliced confocal image shows the cross-section of skin under the confocal laser microscope. For both controls, it can be seen that the drug mainly resides within the first 100 µm of the skin, which comprised stratum corneum and epidermis. On the other hand, proposome was able to deliver the drug to much deeper regions of the skin. It can also be seen that drug is moved via the transfollicular pathway and the follicles are shown as dots. The depth of penetration by the drug can also be observed, where proposome showed higher fluorescence intensity at the deeper part of the skin. The depth of drug absorption can also be observed in Supplementary Materials S4, where the proposome showed higher fluorescence intensity at deeper parts of the skin.

Out of five proposome formulations, only the one containing rhodamine B was imaged using confocal microscopy, as other drugs were not fluorescent. Rhodamine B is widely used as a lipophilic fluorescent probe while calcein is widely used as a hydrophilic fluorescent probe. The confocal study with these fluorescent probes represents either hydrophilic molecules or lipophilic molecules. The images show the general comparison of skin penetration of the control vs. proposomes containing hydrophilic or lipophilic small molecules, but there may be variations for specific drugs.

### 3.5. RHS for Toxicity Assessment

**Figure 7:**
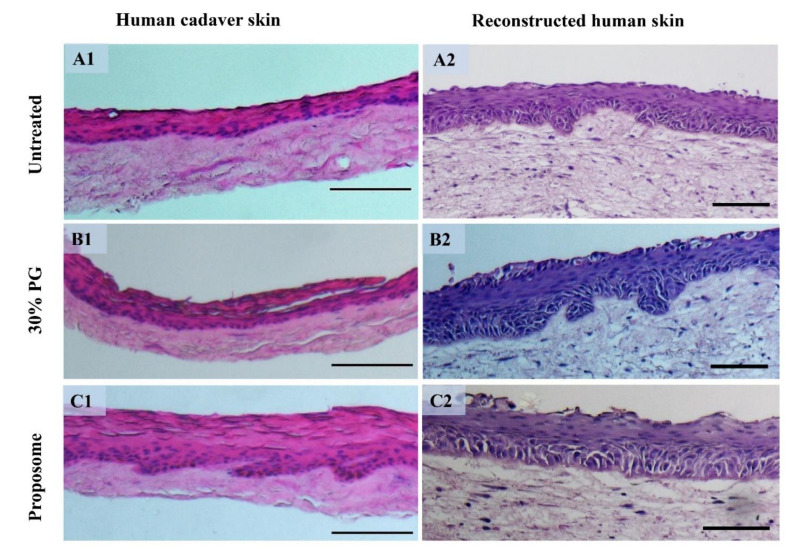
Histological section after hematoxylin and eosin (H&E) staining of human cadaver skin and RHS before and after treatment. (**A1**) Untreated human cadaver skin and (**A2**) RHS. (**B1**) Human cadaver skin and (**B2**) RHS treated with 30% PG. (**C1**) Human cadaver skin and (**C2**) RHS treated with blank proposome. (Scale bar = 100 µm).

We have developed a RHS model using hydrogels, embedded with human endothelial cells, vascular smooth muscle cells, fibroblasts, and keratinocytes. The prepared 3D skin tissue had distinct epidermis and vascularized dermis, which mimic the native skin (Figure 7). It could provide similar permeability and pharmacological response to chemicals, like native human skin [37,51]. The RHS provided a useful model to assess toxicity based on measures of inflammatory markers.

Conventional 3D tissue constructs, majorly collagen-based skin equivalents, undergo scaffold contraction due to biomaterial property. Such contractile actions inhibit cell motility and growth, especially in the case of vascularization. To address such issues, we have developed a method for fabricating multicellular stratified 3D skin tissue equivalents by seeding cells in PEG-Fibrin rich extracellular matrix (ECM) [37]. The vascularized 3D skin constructs did not show tissue contraction and mimicked natural ECMin human skin. Using a similar approach, we also fabricated functional 3D vascularized beds by co-culturing pericytes and endothelial cells derived from pluripotent stem cells. The construct eventually formed lumenized microcapillary structures under a chemically defined growth medium [52].

In bioengineering RHS, addition of pericytes can influence dermal microvasculature. These perivascular cells possess mesenchymal stem cell like characteristics, besides their ability to be active in the fibroblast like cells they have the ability to divide into osteogenic, chondrogenic, and even adipogenic lineages. Pericyte co-culture led to increased deposition in the dermal-epidermal junction of laminin-511/521. Particularly, pericytes were found to enhance tissue formation, as compared to dermal fibroblasts [53].

### 3.6. Histological Study

The histological assessment is widely used to visualize the damages and/or anatomical changes as a cause of application of topical products, for example, micropores after microneedle treatment [34,54] and laser treatment [55], increase in vascularization after radiofrequency treatment [56], swelling of skin layers caused by ethanol [20], etc. Herein, the samples did not show any signs of toxicity regarding physical damage or change in skin integrity after treatment for 24 h. The hematoxylin and eosin (H&E) staining results are shown in Figure 7. The slight swelling of epidermis was seen in the proposomes treatment compared to the untreated skin in both cadaver skin and RHS. However, the skin layers are intact in both the cadaver skin and RHS.

### 3.7. Toxicity Assessment of Proposomes

**Figure 8:**
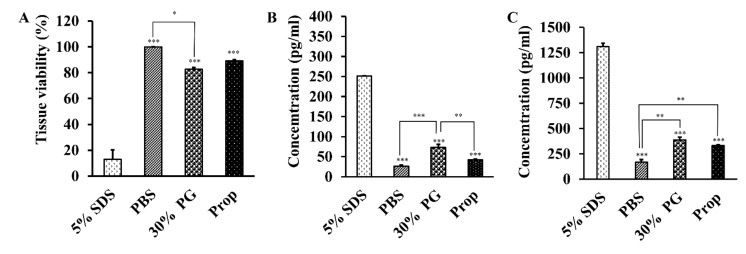
RHS results after treatment for 24 h with PBS, 30% PG, 5% *w/v* SDS, and blank proposome. (**A**) Tissue viability (%) measured using MTS assay. (**B**) IL-α and (**C**) IL-8 levels measured using ELISA kit. (N = 2, * *p* ≤ 0.05, ** *p* ≤ 0.01, *** *p* ≤ 0.001).

The RHS was used as a viable skin model to measure the tissue viability using (3-(4,5-dimethylthiazol-2-yl)-5-(3-carboxymethoxyphenyl)-2-(4-sulfophenyl)-2H-tetrazolium) (MTS) assay (Figure 8A). The MTS assay reflects the cellular functioning (mitochondrial damage) of the cells. As the positive control, the 5% SDS solution significantly reduced tissue viability, compared with PBS and 30% PG and blank proposome. The blank proposome was not significantly different from PBS. The 30% PG lowered the tissue viability significantly, compared with PBS.

In addition to viability, the levels of IL-1α and IL-8 were also measured using the ELISA kit, after the RHS was treated with the four preparations respectively for 24 h (Figure 8B,C). The cytokines of IL-1α and IL-8, are indicative of skin irritation. Similar to the viability results, the 5% SDS solution resulted in significantly higher levels of IL-1α and IL-8 than PBS, 30% PG, and blank proposome. The 30% PG resulted in slightly higher levels of IL-1α and IL-8, compared with PBS. The blank proposomes had minimal elevation of IL-8 level, but not IL-1α level.

The skin irritation and toxicity were assessed in human cadaver skin and RHS. RHS provides a standardized human skin model to test drugs for histology, viability, and cytokine assay, as it is biochemically and physiologically like human skin [57,58,59]. The reconstructed epidermal and dermal skin models, such as EpiDerm, SkinEthic, and EPISKIN, have been used for testing corrosivity, drug permeation, and irritation potentials [57,58,59,60]. The Organization for Economic Co-operation and Development (OECD) test guidelines also endorsed a skin test, namely, TG 431 Human Skin Model Test.

IL-1α and IL-8 are commonly used cytokine markers to assess skin irritation. In human skin, IL-1α is an inherent and biologically active protein present in the epithelial cells that come in contact with the external environment [61]. Similarly, IL-8 is a potential chemokine produced in skin by various cells, including keratinocytes [62]. Hairui et al. used IL-1α and IL-8 as markers of toxicity assay in keratinocytes caused by copper [63]. The low levels of IL-1α and IL-8 found in RHS (Figure 7), confirmed that the proposome was biocompatible as compared to SDS, which resulted in very high levels of IL-1α and IL-8. Furthermore, the histology testing did not show any sign of skin toxicity, despite minimal epidermis swelling. In another study, Llewelyn et al. showed swelling of frog skin when treated with 20% PG, but without causing skin damage, demonstrating the safety profile of PG for skin applications [20].

## 4. Conclusions

We have demonstrated that proposomes can efficiently entrap drugs with different physicochemical properties and improve overall skin permeation. Particle size was uniform for all proposomes, regardless of the drug loaded. Drug entrapment efficiency was correlated with drug logP. Higher entrapment efficiency was positively associated with higher drug permeation and retention. Overall, the proposomes are safe and effective in enhancing drug permeation through the skin. The findings can help formulation scientists better understand drug encapsulation in proposome and its skin permeation to design better topical drug formulations.

## Figures and Tables

**Table 1:** Characteristics of proposomes containing different drugs.

Drug	MW	logP	mp (°C)	ζ (mV)	Size (nm)	PDI	EE (%)	DL (%)
Blank	-	-	-	−28.7 ± 1.06	393 ± 36.5	0.52 ± 0.06	-	-
Ibuprofen	206	3.97	76	−26.9 ± 0.71	145 ± 1.8	0.08 ± 0.06	55.7 ± 5.2	6.5 ± 0.6
Ibuprofen Na	228	0.92	76	−32.5 ± 0.35	141 ± 2.9	0.04 ± 0.01	51.6 ± 6.6	6.65 ± 0.9
Tofacitinib	505	1.15	200	−3.36 ± 0.25	148 ± 1.6	0.13 ± 0.01	42.9 ± 4.4	12.2 ± 1.3
Rhodamine B	479	2.43	210	11.8 ± 0.28	128 ± 1.1	0.14 ± 0.00	52.7 ± 5.3	14.3 ± 1.5
Lidocaine	234	2.44	68	−24.1 ± 0.35	143 ± 1.2	0.11 ± 0.00	54.7 ± 4.5	7.2 ± 0.6

%EE: percentage entrapment efficiency; %DL: percentage drug loading; PDI: Polydispersity index; mp: melting point; ζ: Zeta potential.

**Table 2:** Linear regression of the skin permeation parameters obtained from ibuprofen, lidocaine, tofacitinib citrate, and rhodamine B. Ibuprofen sodium was excluded in the regression analysis The cumulative permeation (CP), CP enhancement (EN_CP_), and CP enhancement ratio (ER_CP_) as dependent variables, and logP, mp and MW as independent variables. The AUC, AUC enhancement (EN_AUC_) and AUC enhancement ratio (ER_AUC_) were not applicable (NA) to total skin permeation.

Total (In Skin + In Receptor Compartment)		In Receptor	
Linear Equation	R^2^	Linear Equation	R^2^
CP = −0.0015 MW + 0.762	0.950	CP = −0.0013 MW + 0.654	0.986
ENCP = −0.0009 MW + 0.479	0.986	ENCP = −0.0009 MW + 0.430	0.916
ERCP = −0.005 MW + 4.152	0.499	ERCP = −0.0063 MW + 4.699	0.501
NA		AUC = −2.9468 MW + 1536.6	0.946
NA		ENAUC = 1.7 MW + 849.41	0.692
NA		ERAUC = −0.0029 MW + 3.049	0.246
CP = −0.0031 mp + 0.665	0.989	CP = −0.0027 mp + 0.562	0.992
ENCP = −0.0019 mp + 0.406	0.950	ENCP = −0.0017 mp + 0.359	0.850
ERCP = −0.0088 mp + 3.573	0.359	ERCP = −0.0111 mp + 3.978	0.364
NA		AUC = −5.9994 mp + 1319.1	0.932
NA		ENAUC = −3.2269mp + 691.51	0.764
NA		ERAUC = −0.0043mp + 2.622	0.130
CP = 0.1271 LogP − 0.077	0.385	CP = 0.1263 LogP − 0.122	0.504
ENCP = 0.1037 LogP − 0.116	0.636	ENCP = 0.1049 LogP− 0.142	0.716
ERCP = 0.9267 LogP + 0.045	0.906	ERCP = 1.1545 LogP − 0.439	0.895
NA		AUC = 308.9 LogP − 284.08	0.557
NA		ENAUC = 243.84 LogP + 364.81	0.593
NA		ERAUC = 0.715 LogP + 0.245	0.823

## Data Availability

Not applicable.

## Supplementary Materials

The following are available online at https://www.mdpi.com/article/10.3390/pharmaceutics13091457/s1. Supplementary Materials S1. Consideration of drug concentration and sink condition. Supplementary Materials S2. High performance liquid chromatography (HPLC) conditions for ibuprofen, tofacitinib citrate, rhodamine B and lidocaine. Supplementary Materials S3. Drug pKa and formulation pH. Supplementary Materials S4. Intensity of confocal laser light at different skin depth.

## Abbreviations

AUC: area under the curve; CLSM: confocal laser scanning microscopy; CP: cumulative permeation; DL: drug loading; EE: entrapment efficiency; ELISA: enzyme-linked immunosorbent assay; EN_CP_: CP enhancement; ER_CP_: CP enhancement ratio; EN_AUC_: AUC enhancement; ER_AUC_: AUC enhancement ratio; H&E: hematoxylin and eosin HPLC: high-performance liquid chromatography; HRP: horseradish peroxidase; mp: melting point; MTS: (3-(4,5-dimethylthiazol-2-yl)-5-(3-carboxymethoxyphenyl)-2-(4-sulfophenyl)-2H-tetrazolium); MW: molecular weight; NA: not applicable; PBS: phosphate-buffered saline; PDI: polydispersity index; PEG: polyethylene glycol; PEVs: penetration enhancing vesicles; PG: propylene glycol; RPM: revolution per minute; RHS: reconstructed human skin; SC: stratum corneum; SDS: sodium dodecyl sulphate; SPC: soya phosphatidylcholine.
