# Supplementary material for: Enhancement of Skin Delivery of Drugs Using Proposome Depends on Drug Lipophilicity
Source: Pharmaceutics. 2021 Sep 13;13(9):1457. doi: 10.3390/pharmaceutics13091457 (PMC8469902; doi:10.3390/pharmaceutics13091457)
Supplement: Supplementary file 1 [file pharmaceutics-13-01457-s001.zip › pharmaceutics-1333778-supplementary.pdf]

pharmaceutics

Supplementary Materials: Enhancement of Skin Delivery of Drugs using Proposome Depends on Drug Lipophilicity

Himanshu Kathuria, Harish K. Handral, Saera Cha, Diep T. P. Nguyen, Junyu Cai, Tong Cao, Chunyong Wu and Lifeng Kang

Supplementary Material S1. Consideration of Drug Concentration and Sink Condition

In this study, the concentration for all drugs was set at 5.6 mM, which is an arbitrary value. During the preparation of proposome, the total amount of drug was mixed with pure PG first. Each drug was easily soluble and formed clear solution at 5.6 mM. Also, the concentration was selected to ensure sink condition in the receptor compartment for skin permeation study.

The calculation of sink condition for each drug is shown in Table S1. For example, the solubility of ibuprofen is 3.4 mg/mL at 37 °C [1]. If 20% of the drug solubility is regarded as the sink condition, the concentration at sink condition is 3400/5 = 680 μg/mL. On the other hand, the amount of ibuprofen added to the donor compartment is 58.17 μg and the maximum possible drug concentration in the receptor compartment (containing 4 mL of PBS pH 7.4) is 58.17/4 = 14.5 μg/mL, which is lower than the sink condition concentration (28 μg/mL). Moreover, 1 mL sample solution was replaced with fresh medium at collection time, further ensuring the sink conditions.

Table S1. Drug solubility and sink condition calculation.

Drug	Solubility in Aqueous Solution (mg/mL)	Amount (μg) in 50 μL of Proposomes	Max. Drug Concentration in Receptor Cell (μg/mL)	Sink Conditions (μg/mL)
Ibuprofen	3.4 (pH 7.4) [1]	58.17	14.5	680
Ibuprofen Na	8.7 (pH 6.8) [1]	64.37	16.1	1740
Tofacitinib Citrate	0.15 (basic) [2]	142.27	35.6	30
Rhodamine B	15 [3]	135.08	33.8	3000
Lidocaine	44.5 (pH 7.4) [4]	66.08	16.5	8900

Supplementary Material S2. High Performance Liquid Chromatography (HPLC) Conditions for Ibuprofen, Tofacitinib Citrate, Rhodamine B and Lidocaine

Table S2. HPLC conditions for the test drugs.

Drugs	Ibuprofen and Ibuprofen Na [5]	Tofacitinib Citrate [6]	Rhodamine B [7]	Lidocaine [8]
Column	ACE 5 C18 column (Advanced Chromatography Technologies, 5 µm, 4.6 × 250 mm)	Zorbax Eclipse XDB C18 column (Agilent, 5 µm, 4.6 × 150 mm)	Zorbax Eclipse XDB C18 column (Agilent, 5 µm, 4.6 × 150 mm)	ACE 5 C18 column (Advanced Chromatography Technologies, 5 µm, 4.6 × 250 mm)
Mobile Phase & Composition	Isocratic Mode 0.05M Sodium Acetate buffer	Gradient Mode A: 0.01 M Ammonium Acetate buffer, pH 5.0 adjusted with Acetic acid. B: Acetonitrile. The gradient is: 0–4.3 min: 75% A and 25% B; 4.31–4.9 min: 5% A and 95% B; and 5.0–10.0 min: 75% A and 25% B)	Isocratic Mode Methonal (75%) Water (25%)	Isocratic Mode 5% Acetic Acid pH=3.4, adjusted with 1.5M NaOH (80%) Acetonitrile (20%)
Detector	UV	UV	Fluorescence	UV
Detection wavelength (nm)	264	287	Excitation: 550 Emission: 580	254
Flow rate (mL/min)	0.8	1	1.1	1.5
Retention time (min)	9.2	4.8	5.2	4.9
Calibration range (µg/mL)	1–200	0.5–500	0.1–25	1–500
Calibration equation and R^2	y = 1990.3x R^2 = 0.9981	y = 39703x − 2618.6 R^2 = 1	y = 2E+07x − 722617 R^2 = 0.9987	y = 1269.4x − 91.271 R^2 = 1

Supplementary Material S3. Drug pKa and Formulation pH

During the process of the proposome preparation, the pH of the solution was not buffered. The final pH of each formulation, i.e., drug laden proposome in 30% PG, has been measured using a glass pH meter and shown in Table S3. PG is a co-solvent, which should be uniformly distributed throughout the continuous aqueous phase of the colloidal system, not particularly localized to lipid bilayer or core of the proposome.

Table S3. The pH of the final proposome formulation and pKa of each drug.

Drug	pH of Final Formulation	pKa	Source
Blank	4.5 ± 0.2	-	-
Ibuprofen	4.3 ± 0.2	4.4	[9]
Ibuprofen Na	6.7 ± 0.1	4.4	[9]
Tofacitinib	4.1 ± 0.2	5.2	[2]
Rhodamine B	3.4 ± 0.2	4.2	[10]
Lidocaine	7.5 ± 0.4	7.9	[4]

Supplementary Material S4. Intensity of Confocal Laser Light at Different Skin Depth

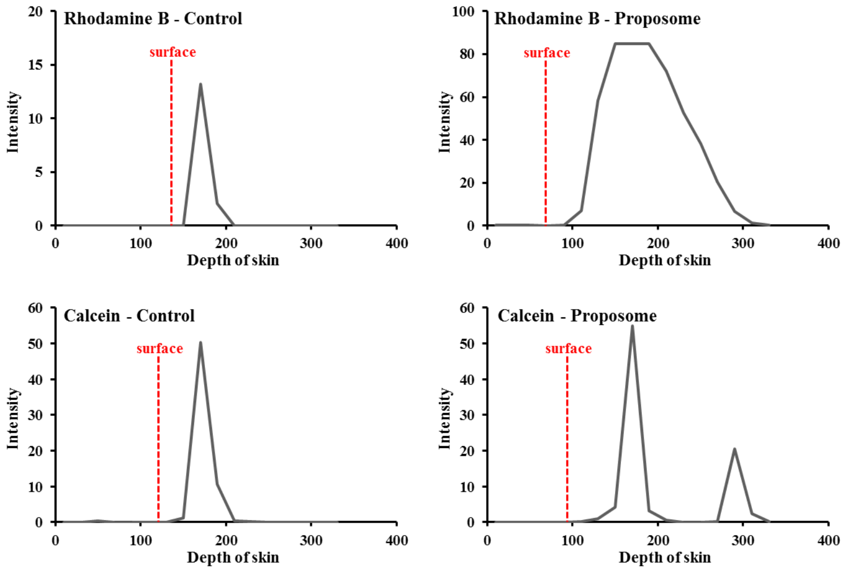

Figure S1. The fluorescent intensity of rhodamine B and calcein inside skin.

References

1. Cristofoletti, R.; Dressman, J.B. Dissolution methods to increasing discriminatory power of in vitro dissolution testing for ibuprofen free acid and its salts. *J. Pharm. Sci.* **2017**, *106*, 92–99, doi:10.1016/j.xphs.2016.06.001.
2. Younis, U.S.; Vallorz, E.; Addison, K.J.; Ledford, J.G.; Myrdal, P.B. Preformulation and evaluation of tofacitinib as a therapeutic treatment for asthma. *AAPS PharmSciTech* **2019**, *20*, 167, doi:10.1208/s12249-019-1377-0.
3. Cheng, C.; Cai, Y.; Guan, G.; Yeo, L.; Wang, D. Hydrophobic-force-driven removal of organic compounds from water by reduced graphene oxides generated in agarose hydrogels. *Angew Chem Int Ed Engl* **2018**, *57*, 11177–11181, doi:10.1002/anie.201803834.
4. Pedersen, B.T.; Larsen, S.W.; Ostergaard, J.; Larsen, C. In vitro assessment of lidocaine release from aqueous and oil solutions and from preformed and in situ formed aqueous and oil suspensions. Parenteral depots for intra-articular administration. *Drug Deliv.* **2008**, *15*, 23–30, doi:10.1080/10717540701828657.
5. Chen, H.; Chang, X.; Du, D.; Li, J.; Xu, H.; Yang, X. Microemulsion-based hydrogel formulation of ibuprofen for topical delivery. *Int. J. Pharm.* **2006**, *315*, 52–58, doi:http://dx.doi.org/10.1016/j.ijpharm.2006.02.015.
6. S, V.K.; Dhiman, V.; Giri, K.K.; Sharma, K.; Zainuddin, M.; Mullangi, R. Development and validation of a RP-HPLC method for the quantitation of tofacitinib in rat plasma and its application to a pharmacokinetic study. *Biomed. Chromatogr.* **2015**, *29*, 1325–1329, doi:10.1002/bmc.3426.
7. Qi, P.; Lin, Z.; Li, J.; Wang, C.; Meng, W.; Hong, H.; Zhang, X. Development of a rapid, simple and sensitive HPLC-FLD method for determination of rhodamine B in chili-containing products. *Food Chem.* **2014**, *164*, 98–103, doi:http://dx.doi.org/10.1016/j.foodchem.2014.05.036.
8. Zhang, Y.; Brown, K.; Siebenaler, K.; Determan, A.; Dohmeier, D.; Hansen, K. Development of lidocaine-coated microneedle product for rapid, safe, and prolonged local analgesic action. *Pharm. Res.* **2012**, *29*, 170–177.
9. Nerurkar, J.; Beach, J.W.; Park, M.O.; Jun, H.W. Solubility of (+/-)-ibuprofen and S (+)-ibuprofen in the presence of cosolvents and cyclodextrins. *Pharm. Dev. Technol.* **2005**, *10*, 413–421, doi:10.1081/pdt-54446.
10. Yu, Y.; Murthy, B.N.; Shapter, J.G.; Constantopoulos, K.T.; Voelcker, N.H.; Ellis, A.V. Benzene carboxylic acid derivatized graphene oxide nanosheets on natural zeolites as effective adsorbents for cationic dye removal. *J. Hazard. Mater.* **2013**, *260*, 330–338, doi:10.1016/j.jhazmat.2013.05.041.
